# Optimisation of a High-Throughput Model for Mucus Permeation and Nanoparticle Discrimination Using Biosimilar Mucus

**DOI:** 10.3390/pharmaceutics14122659

**Published:** 2022-11-30

**Authors:** Leah Wright, Timothy J. Barnes, Paul Joyce, Clive A. Prestidge

**Affiliations:** UniSA: Clinical and Health Sciences, University of South Australia, Adelaide 5000, Australia

**Keywords:** mucus, nanoparticles, PLGA, mucopermeation, transwell, biorelevant

## Abstract

High-throughput permeation models are essential in drug development for timely screening of new drug and formulation candidates. Nevertheless, many current permeability assays fail to account for the presence of the gastrointestinal mucus layer. In this study, an optimised high-throughput mucus permeation model was developed employing a highly biorelevant mucus mimic. While mucus permeation is primarily conducted in a simple mucin solution, the complex chemistry, nanostructure and rheology of mucus is more accurately modelled by a synthetic biosimilar mucus (BSM) employing additional protein, lipid and rheology-modifying polymer components. Utilising BSM, equivalent permeation of various molecular weight fluorescein isothiocyanate-dextrans were observed, compared with native porcine jejunal mucus, confirming replication of the natural mucus permeation barrier. Furthermore, utilising synthetic BSM facilitated the analysis of free protein permeation which could not be quantified in native mucus due to concurrent proteolytic degradation. Additionally, BSM could differentiate between the permeation of poly (lactic-co-glycolic) acid nanoparticles (PLGA-NP) with varying surface chemistries (cationic, anionic and PEGylated), PEG coating density and size, which could not be achieved by a 5% mucin solution. This work confirms the importance of utilising highly biorelevant mucus mimics in permeation studies, and further development will provide an optimal method for high-throughput mucus permeation analysis.

## 1. Introduction

The development of new pharmaceutical compounds for oral delivery is becoming increasingly challenged by the poor solubility and poor permeability of molecules from the drug development pipeline [1]. Particularly, the poor intestinal permeability of new pharmaceutical compounds significantly limits their clinical translation when a systemic effect is required. It is therefore important that we have efficient, high-throughput models for screening the permeation of these compounds through the gastrointestinal epithelium in a timely manner. For this, a wide variety of cell-based models are routinely used to characterise permeability through the epithelium, however minimal utilisation of models predicting permeability of the overlying mucus layer is a key limiting factor for the development of oral formulations [2]. In addition, current models generally employ mucus mimics that provide an insufficient representation of native mucus, or rely on the use of excised animal mucus which poses ethical concerns [3].

The complex gastrointestinal mucus layer consists of a mixture of water, mucin protein, salts, lipids and cellular debris, forming a viscous crosslinked gel with a multidimensional mesh network and presents both chemical and physical barriers to absorption [4,5]. The replication of these complex characteristics in an in vitro setting has been challenging. The most commonly utilised mucus mimic is a simple 5% solution of the mucin protein, the predominant glycoprotein comprising the mucus layer [6]. While this mimic replicates the native protein concentration, extraction and processing of this protein from native mucus for commercial sale results in the breakdown of intermolecular disulfide bridges and a loss of gel-forming capacity [7,8]. Subsequent reconstitution of this purified protein forms a low-viscosity solution with minimal replication of the original mucus characteristics. For this reason, freshly excised native mucus (from human or porcine intestines) is preferred as a model, however ethical constraints surrounding animal use limit its application [9]. In order to bridge this gap, Boegh et al. (2013) [10] developed a biosimilar mucus (BSM) mimic, employing endogenous quantities of protein, lipid and salts coupled with a rheology modifying polymer which could replicate the rheological behaviour (viscoelasticity and shear thinning properties) of porcine intestinal mucus. Despite its availability, this mimic is underutilised in in vitro testing.

Previously we have demonstrated the ability of anionic poly (lactic-co-glycolic) acid (PLGA) nanoparticles to permeate a 5% mucin solution with minimal hindrance compared to free FITC-OVA protein in a novel microfluidic device. Despite this, the permeation was hindered 5.5 fold when tested using the more biorelevant BSM [11]. This was attributed to the limited electrostatic binding interactions between anionic PLGA nanoparticles and mucin fibres (containing a large proportion of anionic sialic acid residues) [12,13]. In contrast, the utilisation of a more biosimilar mucus mimic clearly presented additional barriers including hydrophobic interactions, size-exclusion effects and significantly increased viscosity, resulting in reduced permeation more reflective of the native gastrointestinal environment [14]. While the microfluidic model applied for this analysis is highly valuable for exploring interactions with mucin fibres on a microscopic level, the low throughput and propensity for leakage of high viscosity solutions necessitates an alternate approach for initial screening.

The application of BSM, with a known composition of proteins and lipids, for permeation studies provides further significant benefits in the case of acid or enzyme labile drugs. With native mucus comprising an unknown, variable composition with likely retention of intestinal contents including digesta and proteolytic enzymes, the analysis of mucus permeability for these compounds may be confounded by concurrent degradation [15]. One such class of compounds are biologics, including proteins and peptides, which are increasingly utilised for their high potency and specificity [16]. Despite this, their delivery remains predominantly parenteral and their translation to the oral delivery route is still challenged by their poor permeability and high susceptibility to enzymatic cleavage [17]. Their clinical translation has therefore often been coupled with nanoparticle carriers to protect them and efficaciously deliver them to the target site [18]. This has been the case in many vaccines, including the recently approved lipid nanoparticle-based COVID-19 vaccines by Moderna and Pfizer, as well as virosome based vaccines for Hepatitis A and Influenza [19]. Nanoparticles may also assist the delivery of biologics via the oral route, protecting them from gastrointestinal pH extremes and enzymes, as well as enhancing mucus and epithelial permeation. These systems are hindered to a variable extent in permeation due to the physical constraints of the mucus mesh size, as well as binding interactions with mucus constituents [20]. Therefore, it is imperative that we have optimised mucus models employing biorelevant mucus mimics to predict how these particulate systems will facilitate transport of their cargoes across the intestinal epithelium.

In this study, we develop an optimised, high-throughput permeation model of the gastrointestinal mucus layer employing a synthetic biosimilar mucus mimic. The model was validated against native porcine intestinal mucus (PIM) with a series of fluorescein isothiocyanate-dextrans (FITC-DEX) of varying molecular weights as model biologics/proteins. Following validation, we explored the ability of BSM to differentiate the permeation of physicochemically diverse poly (lactic-co-glycolic) acid nanoparticle formulations. To our knowledge, this is the first utilisation of biosimilar mucus for the permeation of large protein molecules, resulting in identification of key benefits in comparison to mucin solutions. Furthermore, the analysis of nanoparticle permeation utilising biosimilar mucus is in its infancy. The optimisation of this high-throughput model for mucus permeation with a biorelevant mucus mimic provides significant benefits for the quantification of mucus permeation for large-size therapies such as proteins and nanoparticle formulations.

## 2. Materials and Methods

### 2.1. Materials

2-(N-Morpholino)ethanesulfonic acid (MES), Mucin (Type III) from porcine stomach, cholesterol, bovine serum albumin (BSA), 4 kDa fluorescein isothiocyanate-dextran (FITC-DEX), poly vinyl alcohol (PVA, MW 50-190 kDa), DL-α-Tocopherol polyethylene glycol 1000 succinate (TPGS), magnesium sulfate, calcium chloride, Pluronic F127 (PLUF127) and Corning^®^ Transwell^®^ cell culture inserts (24 well, polycarbonate, 8 µm pore size) were purchased from Sigma Aldrich (North Ryde, NSW, Australia). Tween 80, Sodium chloride and cetyltrimethylammonium bromide (CTAB) were obtained from Chem-Supply (Gillman, SA, Australia). Lipoid S 100 (PC) was gifted from Lipoid GmbH (Ludwigshafen, Germany). Carbopol 974 PNF (PAA) was purchased from Lubrizol (Wickliffe, OH, USA). Poly(lactic-co-glycolic) acid (PLGA, MW 45 kDa, 50:50) was obtained from Hangzhou Dayangchem Co. Ltd. (Hangzhou, China). Ethyl acetate was sourced from Merck Millipore (Bayswater, VIC, Australia). Fluorescein isothiocyanate ovalbumin (FITC-OVA) was synthesized by Dr Shasha Rao (University of South Australia, Adelaide, SA, Australia).

### 2.2. Synthesis of PLGA Nanoparticles with Varying Surface Properties

Three PLGA based nanoparticle systems were prepared for FITC-OVA encapsulation with varying surface properties according to the double-emulsion method. PLGA-NP with an anionic surface charge (hereafter labelled PLGA-NP(-)) were prepared according a previously published protocol, with modifications [21]. 0.5 mL of aqueous FITC-OVA solution (5% *w*/*w* to PLGA) was combined with 2 mL of 10 mg/mL PLGA in ethyl acetate solution and sonicated (Vibra-cell, Sonics&Materials, Newtown CT) on ice for 2 min (80% amplitude, 20 sec pulse) in order to form the primary emulsion. The emulsion was combined with 12 mL of 1% PVA and 0.1% TPGS and the sonication repeated. This secondary emulsion was transferred dropwise to 25 mL of 0.3% PVA and 0.03% TPGS while stirring and the solvent allowed to evaporate overnight. Resultant particles were collected and washed twice with Milli-Q water via centrifugation (28,000× *g*, 15 min, 4 °C). PLGA-NP with a cationic surface charge (hereafter labelled as PLGA-NP(+)) were produced via the above method with substitution of the primary surfactant solution with 1% CTAB, secondary surfactant solution as 0.3% CTAB and PLGA concentration as 30 mg/mL). Preparation of larger nanoparticles for size-dependent permeation analysis was facilitated via the substitution of the second sonication step with high-speed homogenisation (Miccra D-1, Mullheim, Germany), at 14,000 s^−1^ for 3.5 min prior to addition to the second surfactant solution and overnight evaporation.

#### 2.2.1. PEGylation of Anionic PLGA Nanoparticle Surface via PLUF127 Adsorption

PEGylation of PLGA-NP(-) was performed via PLUF127 incubation and based on previous work by Yang et al. (2011) [22] and hereafter labelled PEG-PLGA-NP. PLUF127 concentrations relative to the PLGA quantity initially formulated of 0%, 10%, 20%, 50%, 100%, 200%, 400% and 600% were tested in triplicate. Briefly, PLGA-NP(-) were redispersed in 10 mL Milli-Q water. A 1 mL aliquot of nanoparticle dispersion was combined with the appropriate amount of PLUF127 and shaken for 15 min. Samples were centrifuged (28,000× *g*, 15 min, 4 °C) and the supernatant separated for further analysis. The nanoparticle pellet was redispersed in 10 mM MES buffer and analysed for zeta potential via electrophoretic mobility (Zetasizer Nano, Malvern Panalytical, Malvern, UK).

In the absence of adsorbed PLUF127, particle zeta potential (*ζ*_1_) can be related to surface potential (*ψ*_0_) via a model developed by Fleer (1971) [23], as employed by Barnes et al. (2000) [24].

(1) $$tanh\frac{ze\zeta_{1}}{4kT}=tanh\frac{ze\psi_{0}}{4kT}e^{-k\Delta }$$

where *z* is the valence of potential-determining ions, *e* is the electron charge, Δ is the distance from the particle surface to the shear plane, *k* is the reciprocal Debye length and T is the temperature in Kelvin. Therefore, in the presence of adsorbed polymer, the observed zeta potential (*ζ*_2_) can be related to the adsorbed layer thickness (δ) by Equation (2).

(2) $$tanh\frac{ze\zeta_{2}}{4kT}=tanh\frac{ze\psi_{0}}{4kT}e^{-k\delta }$$

#### 2.2.2. Quantification of Adsorbed PLUF127 Mass

PLUF127 adsorbed mass quantification was determined indirectly via a cobalt thiocyanate assay [25]. The cobalt thiocyanate reagent was prepared via combination of 3 g cobalt nitrate and 20 g ammonium thiocyanate in 100 mL water. Following PLUF127 incubation with PLGA-NP(−), samples were centrifuged (28,000× *g*, 15 min, 4 °C) to pellet the nanoparticle fraction with adsorbed PLUF127. From this, the supernatant containing unadsorbed PLUF127 was separated and used for subsequent indirect analysis of the adsorbed fraction. Unadsorbed PLUF127 was quantified via combination of 200 µL of the supernatant with 200 µL of ethyl acetate and 100 µL of cobalt thiocyanate reagent and the obtained mixture was vortexed and centrifuged (11,500× *g*, 8 min). Non-adsorbed PLUF127 complexed with the cobalt thiocyanate reagent was recovered as a pellet. The supernatant was discarded and the pellet washed with 200 µL ethyl acetate until the supernatant was clean. The resultant pellet was dissolved in 1 mL of acetone and analysed for absorbance at 633 nm. Quantity of non-adsorbed PLUF127 was calculated from standard PLUF127 concentrations prepared via the same method, and utilised to determine adsorbed mass via the difference method.

### 2.3. Transwell Experiments

#### 2.3.1. Preparation of Mucus Mimics

Three mucus mimics were prepared for analysis:

- A 5% mucin solution was prepared via solvation of Type III mucin in MES buffer.
- BSM was prepared via a previously published method [14,26]. Briefly, 5% mucin and 0.9% PAA were dissolved in pH 6.5 MES buffer, with the addition of NaOH to rectify pH. Next, 3.1% BSA, 0.15% Tween 80, 0.36% cholesterol 0.18% (*w*/*v*) PC were added to the mixture and stirred until homogeneous. The prepared BSM was stored overnight at 4 °C prior to use.
- Native porcine intestinal mucus (PIM) was harvested from the intestine of deceased pigs obtained from a local slaughterhouse. Mucus collection was conducted within 30 min from slaughter and kept on ice until snap freezing with liquid nitrogen within 2 h from collection. Permission was gained from the slaughterhouse for the use of waste materials in scientific testing and therefore ethical approval was not necessary for this study.

#### 2.3.2. Permeation Experiments

Mucus mimics were added to 24 well Transwell inserts 30 min prior to the experiment and allowed to equilibrate at 37 °C. The lowest volume of mucus that would give a consistent layer without displacement by addition of the donor solution was chosen. Due to viscosity differences influencing volume dispensed, 50 µL of 5% mucin solution was used, whereas 80 µL of BSM or PIM was used, to give a layer of comparable thickness. Mucus layer thickness was quantified via Image J at 1.26 ± 0.12 mm, 1.24 ± 0.18 mm and 1.28 ± 0.13 mm for 5% mucin, BSM and native PIM, respectively. Prior to the commencement of the experiment, the receiver chamber was filled with 600 µL of MES buffer pH 6.5. Donor solutions were prepared in MES buffer equivalent to 100 µg/mL FITC-OVA or FITC-DEX, and 150 µL was added to the donor chamber at the commencement of the experiment. At 1, 2, 3, 4 and 6 h 50 µL was removed from the receiver chamber and replaced with fresh buffer. At the conclusion of the study all samples were analysed for permeated fluorescence associated with FITC-OVA, and the acceptor chamber analysed for particle number via nanoparticle tracking analysis (NanoSight, Malvern Panalytical, Malvern, UK). Percentage permeation was normalised for each formulation to the quantity of formulation permeating the uncoated Transwell semi-permeable membrane to account for any differences in interaction with the membrane material.

## 3. Results

### 3.1. Fabrication of Nanoparticles with Diverse Surface Properties

In order to test the ability of a high-throughput biosimilar mucus model to discriminate between particle systems with differing muco-permeating abilities, we formulated PLGA-NP with varying surface chemistries, incorporating FITC-OVA as a model protein therapeutic.

Despite the native anionic charge of the PLGA polymer, the final surface chemistry and size of the resultant nanoparticles is dictated by the employed surfactant. All formulations were prepared via the emulsification solvent evaporation method and parameters were optimised to produce nanoparticles of comparable size but differing surface properties. Nanoparticle fabrication utilising polyvinyl alcohol (PVA) and D-α-tocopherol polyethylene glycol 1000 succinate (TPGS) as stabilisers produced particles of 156.6 ± 2.4 nm diameter with an anionic surface charge of −19.0 ± 0.2 mV (Table 1), which have previously shown to be mobile in mucus. In contrast, the mucoadhesive nature of cationic particles has been widely documented due to electrostatic interactions with anionic sialic acid residues in the mucin protein. Therefore, PLGA-NP(+) were formulated as a negative control system via the same method but with incorporation of cetyltrimethyl ammonium bromide (CTAB) as stabiliser. The resultant nanoparticles exhibited a particle size of 170.5 ± 5.2 nm with a cationic zeta potential of 12.1 ± 1.9 mV.

**Figure 1:**
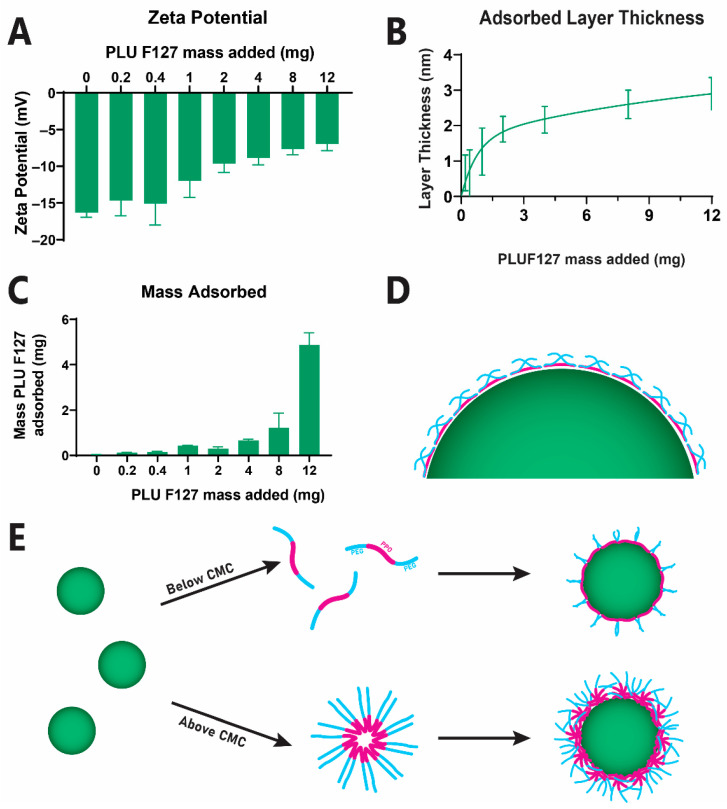
PLUF127 coating of anionic PLGA nanoparticles to provide a physically adsorbed PEG coating layer (**A**) Change in zeta potential following incubation of anionic PLGA nanoparticles with PLUF127 associated with layer thickness (**B**) and resultant mass PLUF127 adsorbed (**C**). Schematic representation of PLUF127 PEG chain extension on the nanoparticle surface (**D**) and mechanism of adsorption (**E**).

PEG-PLGA-NP were prepared via the surface adsorption of PLUF127. Incubation of our PLGA-NP(−) with PLUF127 was found to reduce the magnitude of the zeta potential, i.e., from −16.3 ± 0.3 mV to -7.0 ± 0.9 mV at the highest concentration tested, indicating successful coating with a surface PEG layer and charge-screening (Figure 1A). Furthermore, this increase in zeta potential correlated with an increase in PLUF127 adsorbed mass (Figure 1C). This observation is consistent with the formation of micellar structures in concentrated solutions above the critical micelle concentration of PLUF127 (reported as 0.95-1 mg/mL by the supplier, Figure 1E). The subsequent adsorption of these micellar structures to the particle surface significantly increases the density of the PEG layer formed, resulting in an exponential increase in adsorbed mass in the tested concentration range (Figure 1B) [27]. This is observed with a commencement at 1 mg/mL, indicating that micellar absorption drives the most significant increases in zeta potential and mass adsorbed, whereas monomeric adsorption (below 1 mg/mL) provided negligeable changes in zeta potential due to low mass levels adsorbed to the surface in this form [28].

The thickness of the adsorbed PEG layer was quantified via a simplified electrical double-layer model proposed by Fleer (1971) [23], indicating a layer thickness of 2.9 ± 0.5 nm at the maximum concentration tested here. Considering the geometric size of PLUF127 and the relative length of the PEG segment at 39.6 nm, we can ascertain that the PEG chains extend approximately 9% of their full length into the solution from the particle surface (Figure 1D). This indicates the adsorption of the PEG chains exists in a relatively flat conformation, covering the hydrophobic PPO segments from the aqueous environment [24].

### 3.2. Development and Validation of a High-Throughput Biorelevant Mucus Model

The high-throughput model utilised here was developed based on a Transwell multi-well plate system. For permeation analysis of the large protein molecules and nanoparticle systems, a donor chamber was employed with an associated semi-permeable membrane with pore size of 8 µm to ensure free permeation of the membrane by the protein (model). For mucus permeation analysis, test solutions were added to the donor chamber containing a mucus layer comprised of a 5% mucin solution, BSM or native PIM. Fluorescent signal was quantified in the acceptor compartment to determine the amount of permeated FITC-OVA. In order to account for any differences associated with interaction of the protein or particle systems with the semi-permeable membrane, all samples were normalised to the amount which permeated a blank membrane with no added mucus.

**Figure 2:**
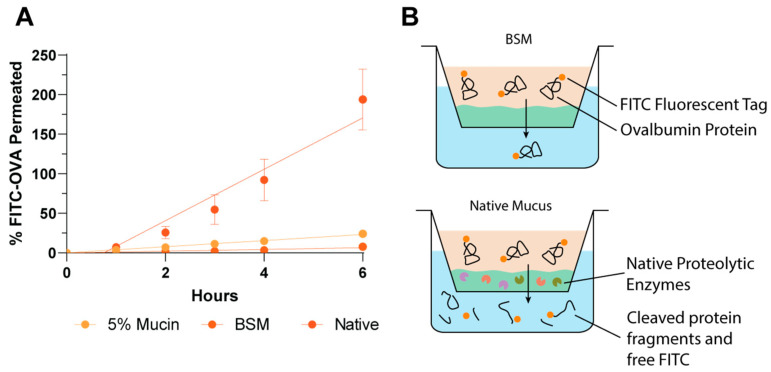
Permeation quantification of free FITC-OVA is incompatible with native mucus. (**A**) FITC-OVA permeation over 6 h detected by FITC fluorescent signal, (**B**) Graphic representation of enzymatic cleavage and free FITC-diffusion in native mucus.

The permeation of free FITC-OVA protein could not be reliably quantified in the native porcine intestinal mucus (PIM) model due to high protease activity resulting in free FITC-dye diffusion and false-positive results (Figure 2). The utilisation of native PIM is an attractive in vitro analysis tool due to the retention of the complete complex natural mucus structure and contents. However, this complex nature may also be to its detriment. The highly variable and unknown content of native PIM causes significant issues for the quantification of fragile therapeutics such as proteins, due to the likely retention of some intestinal contents within the mucus, including enzymes. The proteolytic activity of native gastrointestinal mucus may be attributed to a variety of enzymes, including lysozyme, bacterial or viral secreted enzymes as well as luminal digestion proteases such as gastric pepsin and intestinal trypsin and α-chymotrypsin [29,30,31]. The large quantity of potentially proteolytic species within the unknown chemical mucus mixture significantly increases the complexity of the inhibitor cocktail required to inhibit these, if complete inhibition of all enzymes is possible. The influence of the enzymatic activity of native PIM on the current permeability model for free FITC-OVA analysis is depicted in Figure 2B. The rapid permeation of the small free FITC dye (389 Da) occurs considerably faster than the permeation of intact protein and translates to false-positive fluorescent readings. For this reason, a synthetic mucus mimic which forms a comparable permeation barrier to native mucus is essential for analysis of enzymatically labile compounds.

**Figure 3:**
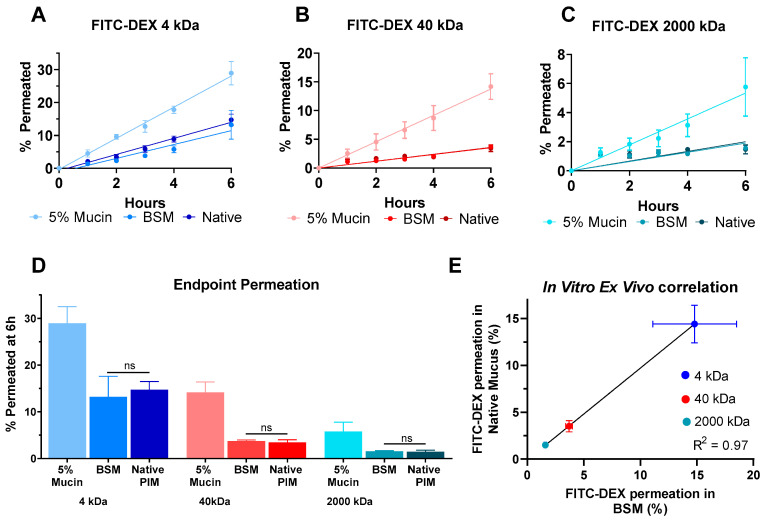
Permeation of 4 kDa (**A**), 40 kDa (**B**) and 2000 kDa (**C**) FITC-DEX as a model protein indicates size and mucus-mimic dependent permeation through a simple Transwell mucus model with efficient modelling of native PIM by BSM. (**D**) FITC-DEX permeation shows no significant difference between native PIM and BSM for all molecular weights at 6 h. (**E**) Excellent correlation (R^2^ = 0.97) is found between native PIM and BSM permeation for increasing size FITC-DEX. All analyses were collected via fluorescent signal of permeated FITC-DEX. Non-significant results are designated ‘ns’.

Due to the incompatibility of FITC-OVA with native mucus, validation of the BSM model against native PIM was conducted with FITC-DEX of differing molecular weights (4 to 2000 kDa) as model protein systems which span a wide range of sizes from 2.8 to 43.5 nm (Figure 3). Utilising a 5% mucin solution, 28.9 ± 3.6%, 14.2 ± 2.2% and 5.8 ± 2.0% of 4 kDa, 40 kDa and 2000 kDa FITC-DEX, respectively, permeated the mucus layer after 6 h of incubation. While this is the most commonly utilised mucus mimic, the 5% mucin solution does not adequately replicate the physical barrier posed by native gastrointestinal mucus due to its low viscosity, resulting in a mixing with the test solution within the donor chamber. The lack of gel-forming capability of commercial mucins is widely documented and attributed to the breakdown of disulphide bridges between protein fibres during processing [7,8,32]. Therefore, while this may provide a useful model of the loosely-adherent mucus layer of the gastrointestinal tract, the more challenging barrier to permeation is the firmly adherent mucus layer which remains epithelium bound.

To address this shortcoming, biosimilar mucus (BSM) has been developed to provide a better model of the natural mucus environment. This preparation has been optimised with a rheology-modifying polymer to increase its viscosity, as well as the addition of other key lipid and protein components of the gastrointestinal mucus in order to more closely replicate the behaviour of the native PIM [10,26]. When utilising this mucus mimic, a defined mucus layer was produced and retained throughout the study and resulted in a total permeation of 13.2 ± 4.4%, 3.7 ± 0.3% and 1.6 ± 0.1% for increasing molecular weight FITC-DEX. This biorelevant mucus mimic provides a significantly higher barrier effect for large molecules, such as dextrans and proteins, regardless of their molecular weight. Furthermore, BSM showed excellent modelling of the native PIM permeation barrier, with no significant differences between permeation values for all molecular weight dextrans in BSM and native PIM (Figure 3D). The application of BSM therefore provides an ideal permeation model which does not pose ethical constraints for animal use, as well as an appropriate model for molecules such as proteins which are labile to enzymatic degradation in the complex native PIM.

### 3.3. Application of a High-Throughput Mucus Model as a Screening Tool to Discriminate between Nanoparticle Mucus Permeability

The ability of this validated high-throughput two chamber mucus model to discriminate between nanoparticle systems of differing mucopenetrating capabilities was assayed with three physicochemically diverse PLGA nanoparticle systems. The development and utilisation of a high-throughput model for efficient rank-ordering of a formulations’ mucopenetrating capacity is a valuable drug-development tool considering the large number of nanoparticulate formulations currently in development for hard-to-treat conditions and therapeutics with poor solubility and permeability. The effective performance of this model will allow for rapid screening of leading formulations, and fast-tracking of efficient mucopermeating systems into late-stage development.

The surface charge of nanoparticle systems can play a key role on their interaction with the mucus layer and underlying epithelium and a significant impact on their ultimate biological fate. PLGA-NP gain their surface charge form the employed surfactant, resulting in highly tuneable surface charge properties. Here, we have analysed the influence of anionic and cationic surface charges via the utilisation of PVA/TPGS or CTAB, respectively. In addition, we analysed PEG-PLGA-NP as the current ‘gold-standard’ for mucopermeation due to their lack of electrostatic interactions with the mucus layer allowing them to slip through the complex mesh structure largely unhindered [33]. These particles were developed to mimic the surface characteristics of viruses, exhibiting a dense array of positive and negative charges on their surface, resulting in a net neutral charge overall [20]. Initial development of virus-like nanoparticles explored the generation of systems with alternating anionic and cationic layers, however this remains exceedingly difficult to accomplish [20]. Alternatively, PEG layers impart a similar charge-shielding behaviour, with extensive research indicating that a dense layer of low molecular weight PEG is optimal for mucopenetration [34]. Yang et al. (2011) [22] explored the adsorption of various poly(ethylene glycol)-poly(propylene oxide)-poly(ethylene glycol) (PEG-PPO-PEG, Pluronic) triblock copolymers to the surface of PLGA nanoparticles to enhance cervicovaginal mucus diffusivity. It was found that a molecular weight of the PPO segment of ≥3 kDa was essential to facilitate sufficient hydrophobic interactions to anchor the polymer to the particle surface, resulting in efficient charge-shielding and particle charges closest to neutral. With the largest PPO and PEG segments, PLUF127-coated particles promoted 280-fold more efficient diffusivity in cervicovaginal mucus, compared to their uncoated counterparts.

**Figure 4:**
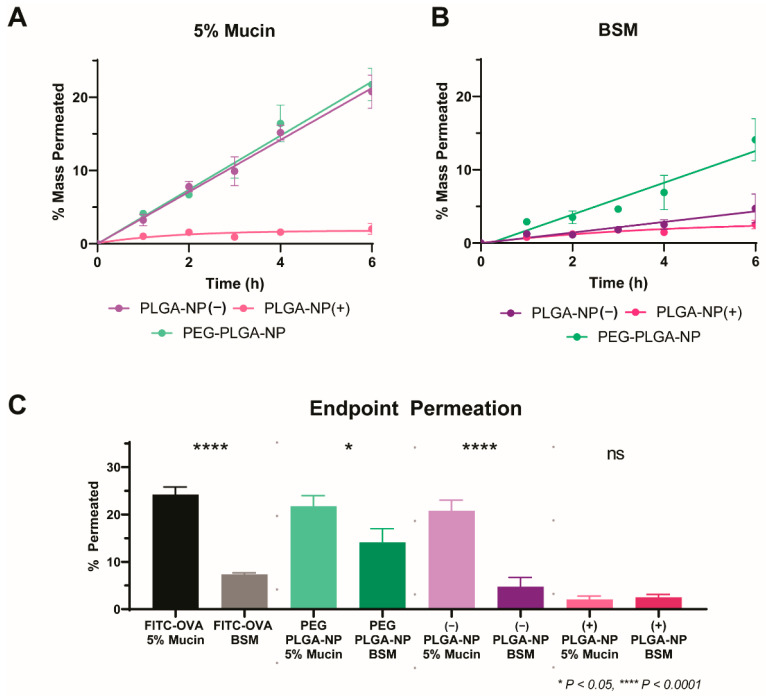
Charge-dependent permeation of PLGA nanoparticles through 5% mucin (**A**) and BSM (**B**). Comparative permeation of all particles and all mucus mimics at 6 h (**C**). All analyses were collected via fluorescent signal of encapsulated FITC-OVA. Non-significant data is designated ‘ns’.

All nanoparticle systems were dosed to the previously validated two-chamber model incorporating mucus layers consisting of 5% mucin or BSM and assayed for permeation over a 6 h period (Figure 4). As anticipated, PLGA-NP(+) showed poor permeation in both mucus mimics. End-point permeation of 2.0 ± 0.7% and 2.5 ± 0.6% of PLGA-NP(+) in 5% mucin and BSM were observed at 6 h, respectively (Figure 3). The comparably low level of permeation of this formulation is attributed to the high degree of electrostatic interaction of the anionic mucin protein with the surface of PLGA-NP(+) [35]. This interaction resulted in a strong immobilisation of the nanoparticles within the mucus layer and prevents any further permeation for both mucus mimics. In contrast, PLGA-NP(−) performed significantly better in 5% mucin, with 20.8 ± 2.3% permeation at 6 h. Furthermore, in agreement with previous data, this permeation was reduced 4.4-fold to 4.7 ± 2.0% when assayed in BSM. As noted in previous work, PLGA-NP(−) permeated with minimal hindrance compared to free FITC-OVA in a 5% mucin solution, and this behaviour was also observed for PEG-PLGA-NP with 21.7 ± 2.2% permeability at 6h. This indicates that no significant interactions of PLGA-NP(−) or PEG-PLGA-NP occurs with the mucin protein. In contrast, permeation of PEG-PLGA-NP through BSM was significantly higher than PLGA-NP(−) at 14.1 ± 2.9% after 6 h. This confirms the significantly increased diffusion of PEG-PLGA-NP through a more viscous, biorelevant mucus mimic.

**Figure 5:**
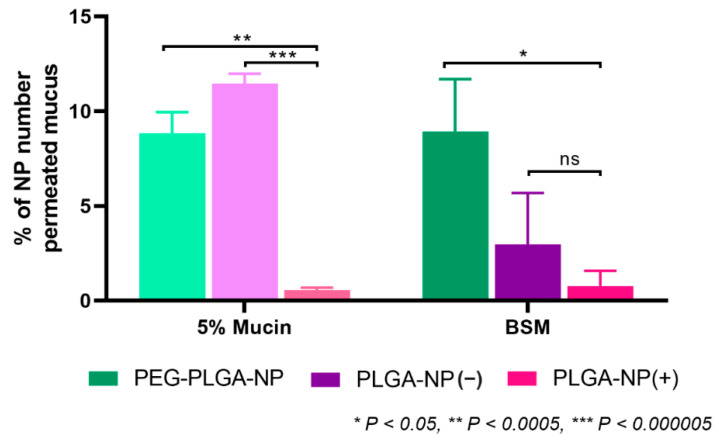
Quantification of the number of basolaterally permeated physicochemically diverse nanoparticles of through model mucus barriers of 5% mucin and BSM after 6 h via Nanoparticle Tracking Analysis (NTA). Permeated nanoparticle number is normalised to the number permeating the bare semi-permeable Transwell membrane without the application of any mucus mimic. Non-significant data is designated ‘ns’.

The enhanced biorelevance of the BSM mucus mimic showed a significantly increased ability for discrimination between the particle permeation of physicochemically diverse nanoparticle systems. In contrast, a 5% mucin solution could not discriminate between permeation of PLGA-NP(−) and PEG-PLGA-NP, both showing a high degree of permeation with no significant difference between the two profiles. BSM however, was capable of efficient discrimination between, and rank-ordering of formulations in order of permeation behaviour. In order to confirm the results observed with fluorescence, particle counting was performed on the basolateral chamber at 6 h via NTA (Figure 5). The observed trends were consistent with the fluorescence readings, confirming that FITC-OVA was retained within the particles and the entire species was transported across the mucus layer. With this approach, the quantification of label-free particle permeation is possible, without reliance on encapsulated fluorescent dye, increasing its applicability to larger volumes of nanoparticle research. For this reason, BSM is identified as a valuable tool for mucus permeation analysis for particulate systems.

### 3.4. Influence of the Degree of PEGylation on Permeation in BSM

**Figure 6:**
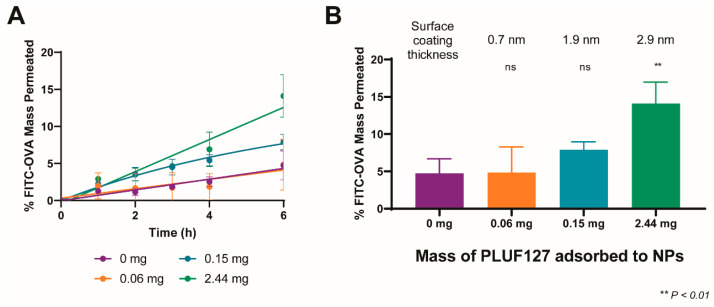
Mucus diffusion of PLGA nanoparticles with increasing degree of PEGylation in BSM (**A**). Comparative permeation of all formulations at 6 h (**B**). Statistics indicate significance compared to uncoated PLGA-NP(−). Non-significant data is designated ‘ns’.

Following validation of the high-throughput model for nanoparticle mucus permeation analysis, we explored the influence of PEG coating density/thickness on the resultant nanoparticle permeation and the ability of our model to discriminate between formulations with different coating levels. For this, we compared the permeation of uncoated nanoparticles (PLGA-NP(−)), the previously analysed PEG-PLGA-NP (coated at the maximal level tested, 2.44 mg adsorbed), as well as two intermediate coating levels, namely 0.06 mg and 0.15 mg of adsorbed PLUF127. The permeation of the particle systems and their encapsulated FITC-OVA cargo was quantitatively analysed from the permeated fluorescence signal over a 6 h period (Figure 6A).

As a 5% mucin solution was previously found to show no capability to differentiate between PLGA-NP(−) and PEG-PLGA-NP, the PEGylated particle variations were tested only in BSM. Adsorption of 0.06 mg of PLUF127 to the nanoparticles resulted in no significant difference in permeation compared to uncoated PLGA-NP(−), with end-point permeation of 5.8 ± 5.1% compared to 4.7 ± 2.0%. Interestingly, the increase in variability associated with the low-density PEG coating indicates that insufficient PLUF127 addition may result in a heterogenous surface coating. The resultant interactions between particles and mucus constituents would therefore be dependent upon the area of the particle surface in direct contact with the mucus layer and its specific surface chemistry. In contrast, increasing the amount of PLUF127 added (and the resultant coating layer thickness) resulted in increasing degrees of permeation, with final permeation at 6 h of 7.9 ± 1.1% and 14.1 ± 2.9% for 0.15 mg and 2.44 mg of adsorbed PLUF127. The adsorption of 0.15 mg PLUF127 was sufficient to facilitate increased permeation of the nanoparticles through the mucus layer, however permeation was still hindered compared to the highest PEG coated particles and not significantly increased over uncoated particles. This increasing trend confirmed the need for high-density PEG layers to facilitate the most efficacious mucus permeation. This can also be attributed to the adsorption mechanism and structure of the PLUF127 during coating, as mentioned previously (Figure 1E). While the high-density PEG layer formed by coating at the highest PLUF127 mass was likely formed due to the presence of micellar PLUF127 structures in solution and their subsequent attachment to the particle surface, the PLUF127 association at lower concentrations was likely monomeric adsorption and significantly less dense.

### 3.5. Size-Dependent Permeation of PEG-PLGA-NP in BSM

A significant benefit of BSM is its complex mesh structure, closely replicating the native mucus mesh, as published previously, that is not observed in the simpler (and more prevalent) 5% mucin solution [11,26]. The nature of this mesh network provides a size-filtering effect and limits the passage of larger molecules and particles to a higher degree than their smaller counterparts. For this reason, we assayed the permeation of PEG-PLGA-NP of two different sizes, the original 188.8 ± 3.4 nm particles, as well as 322.7 ± 4.5 nm particles.

**Figure 7:**
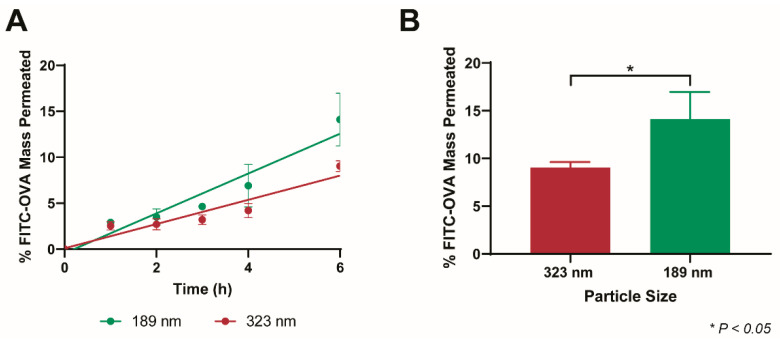
Size-dependent mucus diffusion of PEG-PLGA-NP in BSM (**A**). Comparative permeation of both nanoparticle systems at 6 h (**B**).

PLUF127 coating of 323 nm PLGA-NP(−) was conducted at the maximal coating level previously employed in order to facilitate the adsorption of a similar high-density PEG coating, as proven to be most efficient at mucus permeation. Coating thickness and adsorbed mass were comparable to the smaller nanoparticles, facilitating a similar increase in zeta potential (Table 2). After 6 h, 9.1 ± 0.6% of the 323 nm PEG-PLGA-NP permeated the mucus layer, significantly reduced compared to the 14.1 ± 2.9% associated with the smaller PEG-PLGA-NP (Figure 7). The mesh size of intestinal mucus is widely disputed due to its highly heterogeneous nature, however has been reported to be on the order of 200 nm [36,37]. With this in mind (as well as the thickness of the mucus layer), the overall low permeation of both particle systems is expected. Furthermore, the increased permeation barrier the mucus posed to the permeation of the 323 nm PEG-PLGA-NP highlights the importance of utilising nanoparticle formulations that are as small as possible for effective oral drug delivery (<100 nm ideally). Despite this, particle size reduction is limited by the double-emulsion PLGA-NP fabrication method required to formulate biological molecules (and hydrophilic drugs).

### 3.6. Model Limitations

Nanoparticle permeation across the mucus layer should be rapid enough to achieve complete penetration and contact with the epithelium for subsequent uptake prior to natural mucus turnover. With the complete shedding and replacement of this layer every 47–270 min it is essential that this permeation event is sufficiently rapid [38]. The thickness of the mucus layer employed here (~1.3 mm) is significantly thicker than the natural gastrointestinal mucus layer. The thickness of small intestinal mucus in the rat is stated to be 123–480 µm, however it has been reported to be as low as 30.6 µm. Similar thicknesses have been described in the pig and rabbit, at 26–54 µm and 73–148 µm [39,40]. Human mucus thickness is considerably less studied due to the lack of available tissue, however it has been suggested that the inner adherent mucus layer is of the order of 200 µm, and the loosely adherent mucus layer generally exists as an equal or larger layer [41]. With this in mind, while the current model is effective in comparing between the mucus permeability of formulations, it should not be used to quantify the time required to permeate a natural mucus layer in its current state. Further development of this model to encompass a thinner mucus layer (approximately 500 µm ideally), while retaining the use of BSM for the chemical and rheological replication would be advantageous. This however was limited in the current study via the requirement for a sufficient volume of mucus to withstand the addition of a donor solution, without mucus cratering and disruption of the barrier. The potential exploration of a semi-permeable membrane on top of the mucus layer to strengthen its ability to withstand movement under added solution is considered to be a valuable tool for the use of thinner mucus layers, ensuring it does not influence permeability behaviour.

## 4. Conclusions

This work confirmed the importance of utilising a biorelevant mucus mimic in permeation analysis for reliable discrimination between nanoparticle and protein formulations. We established the benefit of using a synthetic mucus mimic, with known composition, for the analysis of enzyme-labile compounds such as proteins which may experience concurrent proteolytic degradation in native PIM. Furthermore, we confirmed the formation of a comparable permeation barrier between BSM and native PIM using FITC-DEX of various molecular weights. The mucoadhesive nature of PLGA-NP(+) was confirmed with both a 5% mucin solution and BSM. In contrast, significant permeation of PLGA-NP(−) and PEG-PLGA-NP was observed in both mucus mimics. Importantly however, only BSM was able to discriminate between the permeation of PLGA-NP(−) and PEG-PLGA-NP. In addition, the optimised model was capable of the quantification of label-free particle permeation via particle counting in the basolateral chamber. Further characterisation of the model revealed the ability to discriminate between the permeation of nanoparticle systems with increasing PEG coating density, as well as size-dependent permeation. The further development of this model, particularly with respect to mucus layer thickness, will provide a valuable high-throughput method for effective mucus permeation analysis.

## Figures and Tables

**Table 1:** Physicochemical properties of nanoparticles.

Nanoparticle Type	Surfactant	Particle Size (nm)	Zeta Potential (mV)
PLGA-NP(−)	1% PVA + 0.1% TPGS	156.6 ± 2.4	−19.0 ± 0.2
PEG-PLGA-NP	1% PVA + 0.1% TPGS Pluronic F127	188.8 ± 3.4	−7.0 ± 0.9
PLGA-NP(+)	1% CTAB	170.5 ± 5.2	+12.1 ± 1.9

**Table 2:** PLUF127-coating comparison between 189 nm and 323 nm particle systems.

Particle Size	zP (mV)	PLUF127 Coating Thickness (nm)	PLUF127 Mass Adsorbed (mg)
188.8 ± 3.4	−7.0 ± 0.89	2.9 ± 0.4	4.9 ± 0.5
322.7 ± 4.5	−5.0 ± 0.08	4.2 ± 0.1	4.6 ± 0.6

## Data Availability

Not applicable.
